# DNA Repair Biomarkers XPF and Phospho-MAPKAP Kinase 2 Correlate with Clinical Outcome in Advanced Head and Neck Cancer

**DOI:** 10.1371/journal.pone.0102112

**Published:** 2014-07-14

**Authors:** Tanguy Y. Seiwert, XiaoZhe Wang, Jana Heitmann, Vivian Villegas-Bergazzi, Kam Sprott, Stephen Finn, Esther O'Regan, Allan D. Farrow, Ralph R. Weichselbaum, Mark W. Lingen, Ezra E. W. Cohen, Kerstin Stenson, David T. Weaver, Everett E. Vokes

**Affiliations:** 1 Department of Medicine, Section of Hematology/Oncology, The University of Chicago, Chicago, Illinois, United States of America; 2 Department of Surgery, Section of Head and Neck Surgery, The University of Chicago, Chicago, Illinois, United States of America; 3 On-Q-ity Inc., Waltham, Massachusetts, United States of America; 4 Department of Radiation Oncology, The University of Chicago, Chicago, Illinois, United States of America; 5 Department of Pathology, The University of Chicago, Chicago, Illinois, United States of America; 6 The University of Chicago Comprehensive Cancer Center, Chicago, Illinois, United States of America; University of Nebraska Medical Center, United States of America

## Abstract

**Background:**

Induction chemotherapy is a common therapeutic option for patients with locoregionally-advanced head and neck cancer (HNC), but it remains unclear which patients will benefit. In this study, we searched for biomarkers predicting the response of patients with locoregionally-advanced HNC to induction chemotherapy by evaluating the expression pattern of DNA repair proteins.

**Methods:**

Expression of a panel of DNA-repair proteins in formalin-fixed paraffin embedded specimens from a cohort of 37 HNC patients undergoing platinum-based induction chemotherapy prior to definitive chemoradiation were analyzed using quantitative immunohistochemistry.

**Results:**

We found that XPF (an ERCC1 binding partner) and phospho-MAPKAP Kinase 2 (pMK2) are novel biomarkers for HNSCC patients undergoing platinum-based induction chemotherapy. Low XPF expression in HNSCC patients is associated with better response to induction chemoradiotherapy, while high XPF expression correlates with a worse response (p = 0.02). Furthermore, low pMK2 expression was found to correlate significantly with overall survival after induction plus chemoradiation therapy (p = 0.01), suggesting that pMK2 may relate to chemoradiation therapy.

**Conclusions:**

We identified XPF and pMK2 as novel DNA-repair biomarkers for locoregionally-advanced HNC patients undergoing platinum-based induction chemotherapy prior to definitive chemoradiation. Our study provides insights for the use of DNA repair biomarkers in personalized diagnostics strategies. Further validation in a larger cohort is indicated.

## Introduction

Head and neck squamous cell carcinoma (HNSCC) is the 6th most common malignant neoplasm worldwide and accounts for 45.000 new cases in the US every year [1], [2]. For practical purposes, head and neck cancer is divided into three clinical stages: early, locoregionally-advanced, and metastatic or recurrent. Treatment approaches can vary depending on the disease stage. The vast majority of patients (∼60%) presenting with locoregionally-advanced disease require aggressive multimodality therapy. Reported long-term survival rates ranges between 50–70% [3], [4]. Induction or neoadjuvant chemotherapy is increasingly used prior to definitive local therapy (i.e. surgery/chemoradiotherapy/radiation) and FDA approved for this indication. Induction chemotherapy is associated with high response rates, symptomatic relief, and a reduction in distant metastatic failures. Moreover, several groups including ours have reported a clear association between response to induction chemotherapy and improved overall survival [5]–[7]. Despite a high degree of activity, a recent phase III study failed to show benefit of adding induction chemotherapy to chemoradiotherapy in an unselected patient population [8]. Subgroup analysis suggested potential benefit in certain high-risk populations, but in the absence of a suitable biomarker validation of hypotheses will be difficult and expensive.

A meta-analysis also confirmed a small survival advantage with induction chemotherapy despite heterogeneity of the included therapies [9]. Unfortunately, there is currently no validated method to predict which patients will benefit from this therapy and it remains unclear how to select patients for this potentially beneficial as well as potentially toxic therapy. Biomarkers could help to improve patient selection in the future.

DNA repair proteins play an essential role in maintaining genome stability and have been implicated in tumorigenesis. Patients with chromosomal instability syndromes such as Fanconi anemia (FA), ataxia telangiectasia (AT), Bloom's syndrome or Werner syndrome show defects in DNA repair and an associated increased risk and poor prognosis for cancer including head and neck cancers [10]–[17]. Cancer cells exhibit genomic instability and are often defective in one of six major DNA repair pathways namely: base excision repair (BER), nucleotide excision repair (NER), mismatch repair (MMR), homologous recombination (HR), nonhomologous endjoining (NHEJ), and translesion DNA synthesis (TLS). Chemotherapy and most chemotherapeutic agents damage DNA and lack of adequate repair induces tumor cell death.

Therefore, it is crucial to identify DNA repair biomarkers that can predict which patients benefit from induction chemotherapy in locoregionally-advanced head and neck cancer.

Previous reports suggest that ERCC1 is a potential biomarker for platinum-based therapy [18]–[20]. The ERCC1 protein binds to XPF to form a heterodimer, which is a DNA specific endonuclease structure that stabilizes one another *in vivo* and is responsible for the 5′ incision during nucleotide excision repair [21]. Levels of ERCC1 are significantly reduced in XPF deficient cells and vice versa [22]. This biomarker has not been adopted for HNSCC in part due to controversy surrounding the specificity of the employed antibody [23], [24]. Other studies found, that resistance towards platinum-based chemotherapy correlates with protein or mRNA levels of ERCC1 and XPF [21], [25], [26].

In this study, we investigated a panel of DNA repair proteins in five major DNA repair pathways using immunohistochemistry (IHC) and a digital pathology platform to evaluate whether the expression pattern of DNA repair proteins at the biopsy stage can predict tumor response in patients with locoregionally-advanced HNSCC undergoing induction chemotherapy prior to definitive chemoradiation. Our study shows that XPF is highly variable among head and neck cancers with a wide dynamic range: Low levels of expression of XPF correlate with better response to induction chemoradiotherapy, while high levels of XPF expression are associated with a worse response. Furthermore, pMK2, a kinase that has been reported to be critical for post-transcriptional regulation of gene expression as part of DNA damage response [27], is significantly associated with overall survival after induction plus chemoradiation therapy. Our results indicate that the analysis of change in DNA repair pathways may be clinically valuable in HNC.

## Materials and Methods

### Patient cohorts

Biopsy specimens (formalin-fixed, paraffin embedded tumor samples) from 37 patients with stage IV locoregionally-advanced HNSCC treated at the University of Chicago were evaluated from whole sections. The HNSCC patient biopsies had been obtained from a primary excision or biopsy prior to therapy. Written informed consent was obtained from all donors or the next of kin for the use of these samples in research approved under University of Chicago IRB protocol 8980 and 15410A. All patients had been treated with induction chemotherapy consisting of two cycles of paclitaxel and carboplatin for a total of eight weeks. We subsequently performed an interim assessment, followed by paclitaxel, 5-fluorouracil, hydroxyurea and radiotherapy-based regimens (FHX) based chemoradiotherapy and finally we evaluated for response [28], [29]. We analyzed the patient samples regarding their HPV-status by staining for p16 (Santa Cruz JC-8).

### Treatment evaluation

Response evaluation was performed in the interval between induction chemotherapy and consecutive chemoradiotherapy by CT scan and/or clinical examination by an ENT specialist and best response was assessed. Response criteria were defined as complete response (CR) [14], progressive response (PR) and stable disease (SD) based on RECIST criteria [6], [30].

### Cell lines

The simian virus 40-transformed fibroblasts GM08437 (XPF−/−, Coriell Institute) cells and HeLa cells were grown in Dulbecco's modified Eagle's medium supplemented with 10% heat-inactivated fetal calf serum (FCS) in a humidified 5% CO2 incubator at 37°C.

### Immunohistochemistry (IHC)

The whole sections of the samples were stained by IHC using antibodies against XPF (SPM228)/ERCC1 (8F1) (AbCam), FANCD2 (Santa Cruz), PAR, γH2AX (Millipore), MLH1 (AbSerotec) and phospho-MAPKAP Kinase2 (pMK2) (Cell Signaling Technology). Tissue sections were deparaffinized/rehydrated using standard techniques. Heat-induced epitope retrieval was performed and the tissues were stained with antibodies overnight at 4°C. Primary antibodies were omitted for negative controls. Hematoxylin was used as nuclear counterstain. Two-fold antibody dilution ranges were established, and antigen retrieval conditions were set such that antibody was in excess and discriminated between control cancer tissues and between low and high expression levels. Renaissance TSATM (Tyramide Signal Amplification) Biotin System (Perkin Elmer) was used for detection of XPF and FANCD2. Super Sensitive TM IHC Detection System (BioGenex) was used for detection of PAR, PARP1, MLH1, pMK2, γH2AX and ERCC1.

### IHC Scoring

The IHC stained tissues on the slides were scanned into a digital pathology platform (Aperio). Quality of staining pattern was pathology reviewed. Intensity of nuclear staining, and/or localization of the marker into both nuclear and cytoplasmic compartments was determined. Three tumor regions of interest in a whole section were selected by pathologists in order to minimize the effects of IHC staining variation. Scanned slides were then evaluated by pathologists and machine-based digital image analysis (Aperio). The percentage (0–100%) of tumor cells with positive staining Quantity (Q) and intensity (I) for each marker were independently scored by two trained pathologists (VVB, SF), who were blinded from clinical history. A nuclear score was reported for XPF, ERCC1, FANCD2, MLH1, PARP1, PAR and γH2AX. The nuclear and cytoplasmic compartments were scored separately for pMK2. Staining quantity (Q) was scored 0 to 4: no nuclear staining  = 0; 1–9% of cells with nuclear stain  = 1; 10–39%  = 2; 40–69% = 3; and 70–100% = 4. Staining intensity (I) was classified from 0 to 3, with 0 = negative, 1+ = weak, 2+ = intermediate, 3+ = strong. Final scores were obtained by multiplying the quantity and staining intensity scores (IxQ) [31]. Machine-based image analyses were established based on modified macros of the Aperio IHC nuclear algorithm to score the intensity/quantity of positive tumor nuclei. Marker outputs in 0, 1+, 2+, and 3+ bins were combined in a weighting algorithm to create a relative intensity score (H-score) from 0–300 [32].

### Immunoblotting

Immunoblotting for XPF, ERCC1, and β-Actin was done using standard methodology as previously described [33]–[37]. Antibodies used for immunoblotting were anti-XPF (SPM228, AbCam), anti-ERCC1 (8F1, AbCam/Santa Cruz), and anti-β-Actin (H-170, Santa Cruz). Nine head and neck cancer cell lines (SCC58, SCC61, SCC35, SCC28, SQ20B, SCC9, HN5, SCC68, SCC25), kindly provided by Dr. Ralph Weichselbaum and Dr. Mark Lingen, were used.

### Statistical Analysis

Biomarker scoring was correlated with clinical data to assess for correlation with outcome. A set of optimal threshold marker values was determined by univariate analysis for each marker that yielded the highest discrimination to separate Complete response (CR), Partial Response (PR), Stable Disease (SD) groups for induction chemotherapy and overall survival. Multivariate analysis was not feasible due to the small sample number. Univariate Cox proportional hazards models were constructed for each of the markers (single marker models) to examine their potential predictive powers. Discriminant and partition analysis was also conducted to maximally separate the dataset samples into groups.

Statistical outputs for p-value (Positive predictive value), Apparent Error Rate (AER), Receiver Operator Characteristics (ROC) and Area Under Curve (AUC). ROC is a graphical plot of the sensitivity vs. (1-specificity) for a binary classifier system as its discrimination of true positives, in this case, it is 1-specificity (fraction of CR/PR called SD/PD) versus sensitivity (fraction of SD/PD called SD/PD). AUC is a measure of how well two classes of data separate under a testing scheme. Sensitivity, Specificity, Positive Predictive Power, Negative Predictive Power, Relative Risk (RR) and Odds Ratio were computed in the alternative models.

To assess the association of the biomarker scores to overall patient survival, thresholds for each biomarker were determined, which separated patients into two groups. These thresholds were selected by choosing the biomarker value that generated the minimum survival curve p-value when patients with scores above the threshold were compared to patients below the threshold. Thresholds that created a minimum group size of less than 10% of all samples were not considered reliable and excluded from analysis.

Survival curves for the low- and high-risk groups were compared using Kaplan-Meier models and the p-value reported. Additionally, the AER, AUC, ROC curve, sensitivity, specificity, positive predictive value, negative predictive value, and relative risk are reported.

## Results

### Significant variations of DNA repair proteins expression in multiple DNA repair pathways in head and neck cancer

**Figure 1:**
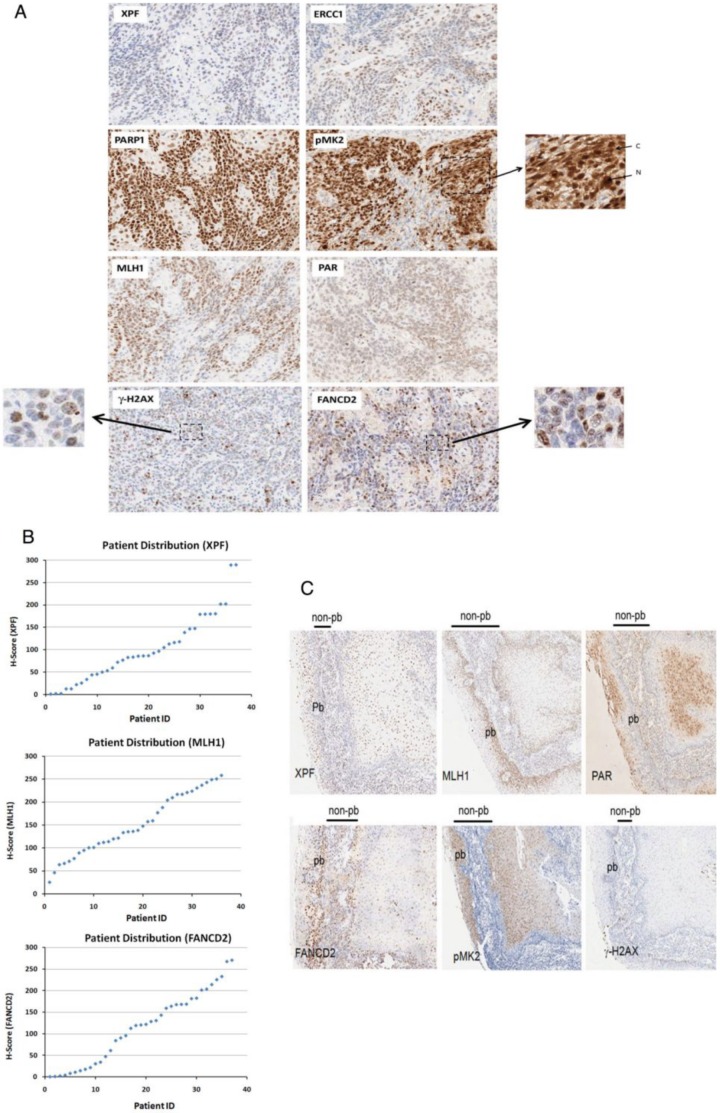
Immunohistochemistry (IHC) staining pattern of the DNA repair biomarkers. A. The FFPE whole sections from 37 HNSCC patient samples were stained by IHC using the antibodies against DNA repair biomarkers (XPF, ERCC1, FANCD2, MLH1, pMK2, PAR, PARP1) according to the protocol described in *Materials and Methods*. The stained tissue on the slide was scanned into a digital pathology platform (Aperio) and images were viewed digitally, magnification 10X. As noted, subcellular localization of pMK2 is in either Nuclear (N), or Nuclear (N) + Cytoplasmic (C), staining patterns of pMK2 in these cancer tissues is shown as indicated, magnification 20X. Nuclear foci in head neck cancer cells were shown for γH2AX and FANCD2 in the lower panel as indicated, magnification 40X. B. Examples of varying biomarker expression in head and neck cancer tissue specimens stained with XPF, FANCD2, MLH1 are shown. Patient distribution of XPF, FANCD2, MLH1 scores are plotted. C. Differences in the staining intensity and distribution of XPF (NER), MLH1 (MMR), PAR (BER), FANCD2 (FA/HR), pMK2 and γH2AX (DDR) in parabasal (pb) and nonparabasal (non-pb) layer cells from specimens of one representative HNSCC patient were shown as indicated.

**Figure 2:**
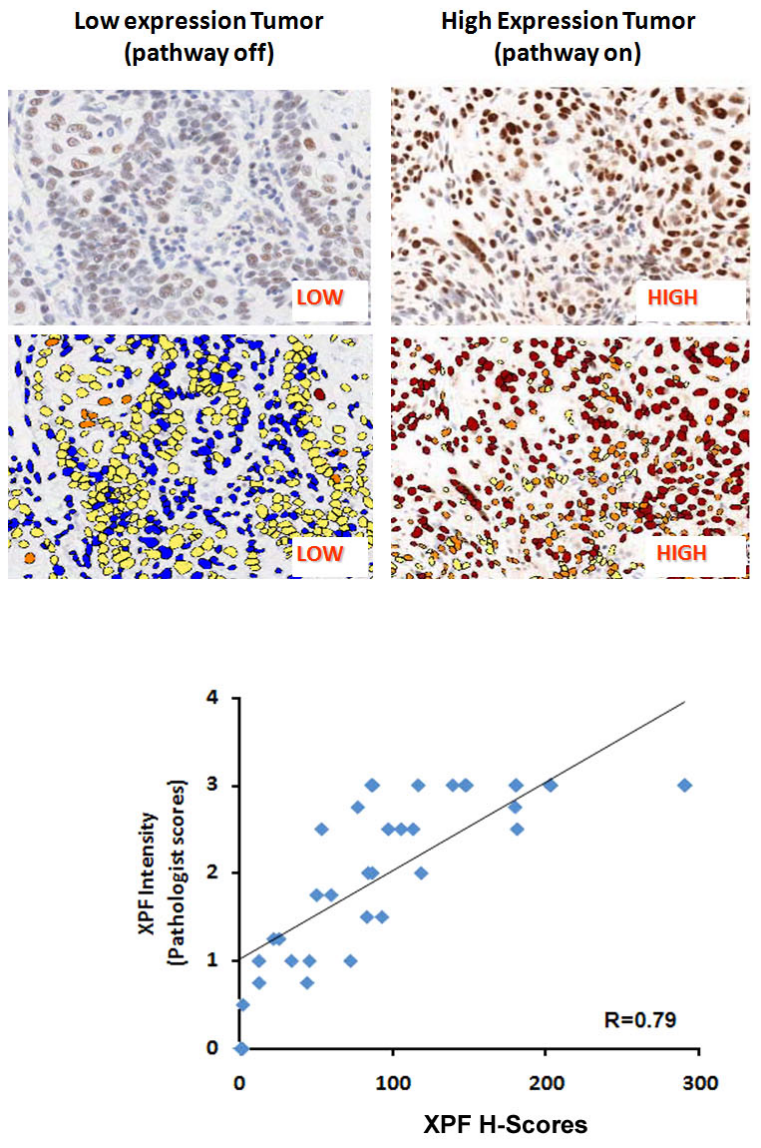
Association of XPF scoring by pathologist scores versus machine assisted image analysis and quantitation. Comparisons are made between alternative scoring strategies for immunohistochemistry with the XPF for each head and neck cancer patient. Machine assisted scoring for XPF was determined based on percentage of nuclei with 1+ (weak), 2+ (medium), 3+ (high) intensity Pathologist scores were Intensity (I). Correlation plots as shown are computed for similarity with an R-value of 0.79.

DNA repair pathways are important for the cellular response to chemotherapy and radiation. Eight selected DNA repair proteins in five major DNA repair pathways were evaluated by IHC in a cohort of 37 patients; an IHC staining example for each biomarker is shown in Figure 1A. Pathologists' scores and machine-based assessment of IHC staining intensities in annotated tumor zones were used to evaluate protein expression differences among patient samples. Expression of DNA repair proteins varies between tumor specimens as shown graphically in the patient distribution for the markers (Figure 1B). Subcellular localization of pMK2 varies between nuclear only, or nuclear + cytoplasmic localization depending on the patient tumor. Several biomarkers such as FANCD2 and γH2AX proteins have a distinct pattern in the nucleus indicative of activation of the FA/Homologous recombination (HR) or DNA Damage Response (DDR) pathway (Figure 1A) in these HNSCC tumors. Biomarkers in different DNA repair pathways such as XPF (NER), MLH1 (MMR), PAR (BER), FANCD2 (FA/HR), pMK2 and γH2AX (DDR) were found to have differences in the nuclear or cytoplasmic staining intensity and distribution between parabasal (pb) and non-parabasal (non-pb) layer cells for certain specimens, suggestive of a variable expression of these DNA repair biomarkers (Figure 1C). An example shown here is the nuclear staining pattern of NER biomarker XPF in two representative cancers by IHC. Low or negative intensity of XPF nuclear staining indicates that NER pathway is off, and high intensity of XPF staining indicates that NER pathway is on (Figure 2). To test the correlations between pathologist scores, machine-guided and image analysis, we compared IHC stained XPF, which were analyzed by two pathologists, who were blinded to tumor samples, and machine-based algorithm in this study (Figure 2) with R2 value of 0.79.

### Highly variable XPF expression in head and neck cancer

**Figure 3:**
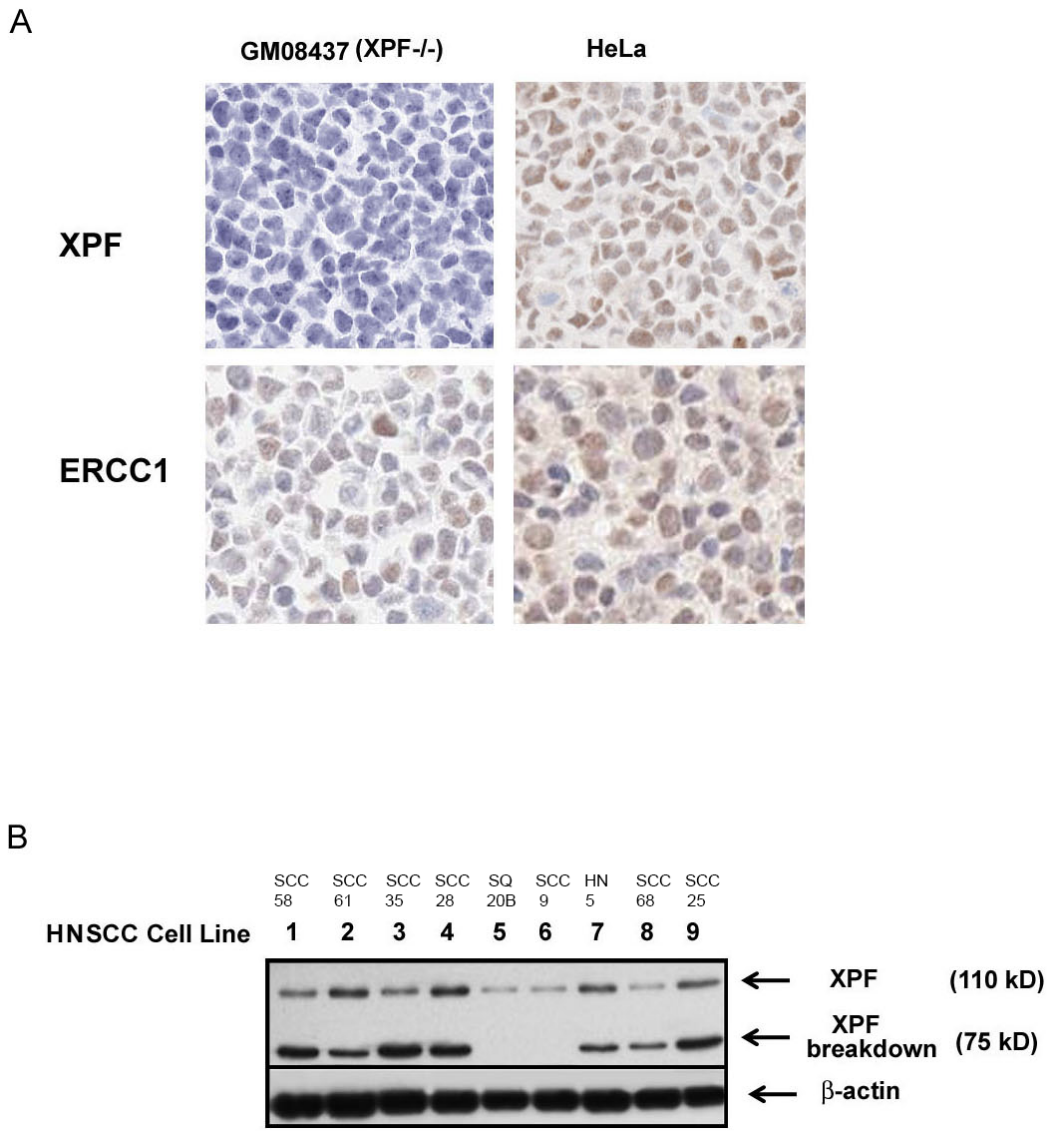
Immunohistochemistry (IHC) staining pattern of XPF and ERCC1 by using anti-XPF (SPM228) and ERCC1 (8F1) antibodies and XPF expression in HNSCC cell lines. A. FFPE blocks of HeLa and GM08437 (XPF deficient cell) pellets were used as negative and positive controls, XPF (SPM228) and ERCC1 (8F1) antibodies were then applied to the sections by immunohistochemistry according to the IHC method for tumor, and nuclear staining patterns of XPF and ERCC1 were shown. B. Nine head and neck cancer cell lines were analyzed by immunoblotting for expression of XPF. XPF and XPF breakdown proteins were detected by an anti-XPF monoclonal antibody (SPM228) with cell lines 5 and 6 showing low expression. β-Actin (Santa Cruz) was used as a protein loading control. The names of the cell lines are listed.

In our study, we determined specificity of the XPF (SPM228) and ERCC1 (8F1) antibodies by IHC using formalin fixed, paraffin-embedded blocks of HeLa (positive control) and XPF deficient cell pellets. Other XPF and ERCC1 antibodies were evaluated (data not shown/proprietary). SPM228 was chosen due to high degree of specificity, and 8F1 chosen as it is the most widely used ERCC1 antibody. Our result showed that specific nuclear staining by a monoclonal antibody against XPF (SPM228) was detected in HeLa cells but not in XPF deficient cells, in contrast, nuclear staining by the ERCC1 8F1 antibody was found in both HeLa and XPF deficient cells, indicating that this SPM228 antibody is XPF specific and suitable for detection of XPF by IHC, and ERCC1 8F1 recognizes additional non-specific nuclear proteins and is unable to specifically detect ERCC1 in specimens (Figure 3A).

We evaluated XPF expression in both, p16(+) and p16(−) samples and did not detect a significant difference (178 versus 165, NS).

We then measured the level of XPF expression in lysates of nine HNSCC cell lines by immunoblot. Two bands of XPF at 110 kD and 75 kD were found consistently, with the 75 kD band recognizing full length XPF and the other band representing a cleavage product of XPF (XPF breakdown) (Figure 3B). We also found that levels of expression of XPF dramatically vary among nine HNSCC cell lines (Figure 3B). A wide dynamic range of XPF expression in the cohort in our study is also shown in a patient distribution plot (Figure 1B). Taken together, our results demonstrate that levels of XPF expression detected by the SPM228 antibody vary significantly in head and neck cell lines and patient specimens, and that the monoclonal antibody SPM228 can be used to specifically detect XPF expression by Western blot and IHC.

### XPF is associated with response to induction chemotherapy for head and neck cancer patients

**Figure 4:**
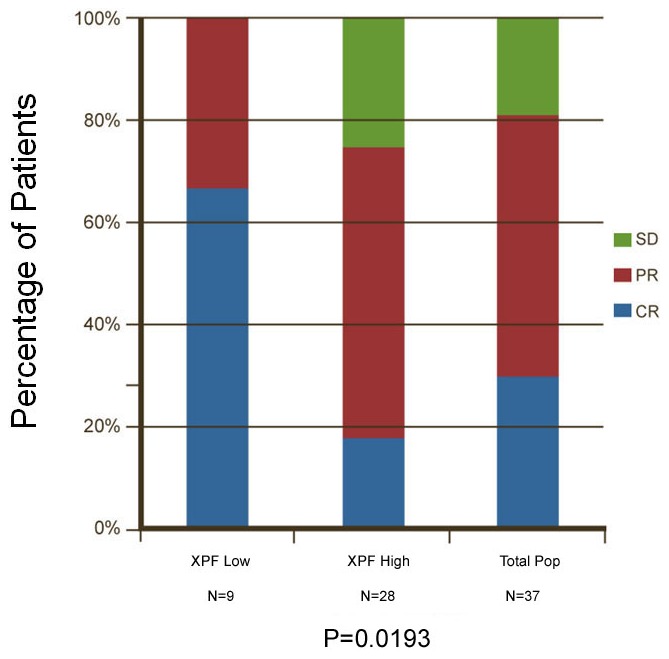
Univariate analysis of XPF biomarker scores shows improved response prediction to induction chemotherapy in head and neck cancer. The chart shows that univariate analysis of the XPF biomarker scores relative to the discrimination between Responder subgroups. The primary outcome measurement was response to induction chemotherapy.

Eight DNA repair biomarkers stained on 37 patient specimens by IHC were analyzed for their ability to predict response to induction chemotherapy. Of the 37 HNC patients treated with induction chemotherapy in the study, complete response (CR) [14] was observed in 11 patients (29.7%), 19 patients (51.4%) obtained a partial response (PR), and seven patients (18.9%) had a stable disease (SD). We found that low levels of XPF expression in HNC patients were significantly associated with better response to induction chemotherapy (p = 0.02) (Figure 4). Moreover, all of seven patients who had SD had high levels of XPF expression (Figure 3, Table 1). In contrast, ERCC1 detected by the commonly used antibody (clone 8F1) in our cohort set did not correlate with response, and other markers such as PARP1, PAR, MLH1, pMK2, γH2AX, FANCD2, also failed to correlate (Table 1). Our results suggest that XPF is the preferred NER biomarker to predict response to induction chemotherapy in HNSCC patients.

**Table 1:** List of DNA repair proteins in univariate analysis of the correlation with response to induction chemotherapy.

Biomarker	ROC plot/AUC value	% Correct Responders at 100% SD/PD Correct	P value (CR/PR vs SD)
XPF	0.783	60	0.0193
ERCC1	0.569	7	0.41
pMK2	0.707	47	0.266
MLH 1	0.545	23	0.616
PARP 1	0.571	20	0.918
PAR	0.509	17	0.872
FANCD2	0.571	13	0.952
γ-H2AX	0.519	N/A	0.629

Higher AUC value means better correlations with response to induction chemotherapy. P values of CR/PR versus SD are shown.

### pMK2 correlates with overall survival to chemoradiation therapy

**Figure 5:**
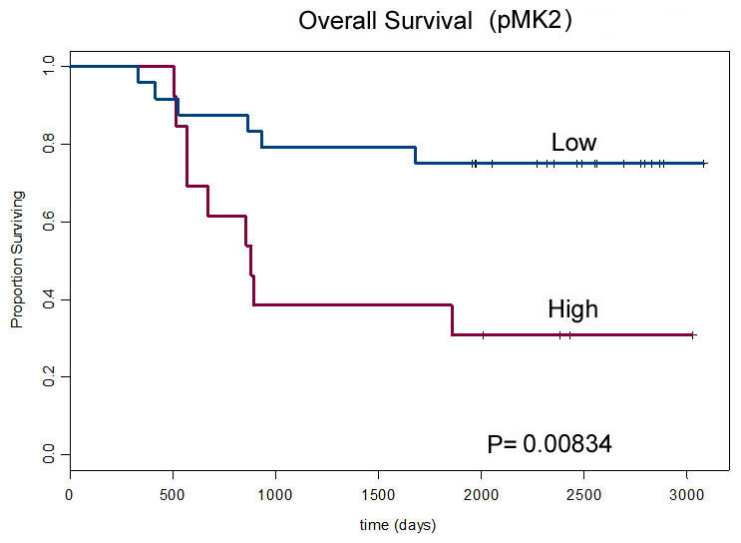
Correlation of expression levels of pMK2 with overall survival. Overall survival estimated by best response to induction chemotherapy using Kaplan-Meier survival curves based on the nuclear staining intensity and quantitation of pMK2 determined by pathologists' scores as NQ (Nuclear Quantity).

We then evaluated association of the DNA repair biomarkers to overall survival for this cohort of patients. pMK2 did not correlate with response to induction chemotherapy (Table 1), but it correlated strongly with overall survival: low pMK2 expression was associated with better overall survival (p = 0.01) (Figure 5); pMK2 differentiated a subgroup with improved survival potentially related to chemoradiation therapy, suggesting that pMK2 may relate to chemoradiation therapy. In contrast, XPF was found not to correlate with overall survival (p = 0.08). For several other markers in DNA repair such as PARP1, PAR, MLH1, γH2AX, ERCC1, FANCD2, the same analysis failed to reach statistical significance (Table 2). Further study of pMK2 is needed.

**Table 2:** List of p values of DNA repair proteins in univariate analysis of the correlation with overall survival.

Biomarker	P value (Overall survival)
XPF	0.0791
ERCC1	0.0873
pMK2	0.00834
MLH 1	0.0474
PARP 1	0.200
PAR	0.141
FANCD2	0.0357
γ-H2AX	0.266

## Discussion

Chemotherapy induces DNA-damage in tumor cells. Therefore the ability to repair such damage using specific DNA repair pathways is likely predictive of drug sensitivity/resistance, and treatment outcome. Thus, diagnostic DNA repair biomarkers hold potential to significantly change diagnostic strategies and affect therapeutic decision-making and treatment planning for patients with head and neck cancer. In our study, we evaluated eight DNA repair biomarkers in five different DNA repair pathways by immunohistochemistry in locoregionally-advanced head and neck cancer. Significant variations in multiple DNA repair pathways were observed in HNSCC tumors suggesting that clinical decisions may be influenced by a DNA repair biomarker profile (Figures 1, 2). Among all of the DNA repair biomarkers that we analyzed, XPF was the single best marker to predict response to induction chemotherapy by univariate analysis; low levels of expression of XPF in head and neck cancer patients were associated with better response to induction chemotherapy. High levels of XPF expression in head and neck patients correlated with worse response to platinum based chemotherapy consistent with prior reports [38]. By contrast ERCC1 (8F1), detected by the commonly used antibody (clone 8F1), in our cohort set did not correlate with response, which may relate to its poorer specificity (Figures 3 and 4, Table 1). ERCC1 (8F1) performance was not adequate in our study and we hypothesize that the decreased specificity can be compensated by larger sample sizes as seen in other studies [18]–[20]. Furthermore it is possible that the ERCC1 8F1 measures something different than ERCC1, which correlates with survival.

While patient response to induction chemotherapy is a potential predictor of good overall outcome as reported by several groups [4], [5], [39]–[41], overall survival remains clinically most meaningful. pMK2 was found to correlate significantly (p = 0.01) with overall survival. Since pMK2 does not appear to relate to induction response it may be a potential marker of treatment success for concurrent chemoradiation (Figure 5, Table 2) consistent with preclinical data [42].

Given the heterogeneity of head and neck cancer, and the intricately connected network of six major DNA repair pathways, it is unreasonable to anticipate that meaningful diagnostic testing can rely on a single, specific marker. As our study suggests, markers for induction and chemoradiation are likely different. Furthermore, compensation of DNA repair in the absence of one repair pathway by another pathway suggests the possibility that multiple markers may be necessary to optimally assess responsiveness. Such a DNA repair response signature will have to be evaluated by our group, using a larger cohort and may allow improved assessment of HNC heterogeneity and complexities of DNA repair networks.

In conclusion, our study provides an established method to measure DNA repair biomarkers and other biomarkers using quantitative immunohistochemistry to identify and evaluate functional changes to DNA repair and damage signaling pathways as a valuable tool for personalized diagnostics. Our results indicate usefulness of XPF as a biomarker to predict which patients benefit from which treatments with induction chemotherapy. Specifically XPF proved superior to ERCC1 (8F1) testing. XPF may also have value to predict overall treatment success, which potentially relates to its role for prediction of induction response [25]. Furthermore, our results suggest that pMK2 is a potential marker for chemoradiation as it did not correlate with induction response, but did correlate strongly with overall survival. Further validation of these markers in a larger cohort of advanced head and neck cancer patients is imperative and our observations are largely hypothesis-forming at this point, but are consistent with other literature [38]. Ultimately, multiple markers may be necessary to optimally assess tumor specimens, and provide the most information to treating physicians.
